# The importance of body composition assessment for patients with advanced hepatocellular carcinoma by bioelectrical impedance analysis in lenvatinib treatment

**DOI:** 10.1371/journal.pone.0262675

**Published:** 2022-01-18

**Authors:** Kenji Yamaoka, Kenichiro Kodama, Tomokazu Kawaoka, Masanari Kosaka, Yusuke Johira, Yuki Shirane, Ryoichi Miura, Shigeki Yano, Serami Murakami, Kei Amioka, Kensuke Naruto, Yuwa Ando, Yumi Kosaka, Shinsuke Uchikawa, Takuro Uchida, Hatsue Fujino, Takashi Nakahara, Eisuke Murakami, Wataru Okamoto, Masami Yamauchi, Daiki Miki, Michio Imamura, Shoichi Takahashi, Akiko Nagao, Kazuaki Chayama, Hiroshi Aikata

**Affiliations:** 1 Department of Gastroenterology and Metabolism, Graduate School of Biomedical and Health Sciences, Hiroshima University, Hiroshima, Japan; 2 Research Center for Hepatology and Gastroenterology, Hiroshima University, Hiroshima, Japan; 3 Cancer Treatment Center, Hiroshima University Hospital, Hiroshima, Japan; 4 Division of Nutrition Management, Hiroshima University, Japan; 5 Collaborative Research Laboratory of Medical Innovation, Hiroshima University, Hiroshima, Japan; 6 RIKEN Center for Integrative Medical Sciences, Kanagawa, Japan; Nihon University School of Medicine, JAPAN

## Abstract

**Background and aims:**

The aim of this study was to investigate the relationship between body composition before lenvatinib treatment and prognosis in patients with hepatocellular carcinoma (HCC). We also assessed the relationship between the rate of change in body composition after lenvatinib treatment and prognosis.

**Methods:**

Eighty-one patients with advanced HCC who were treated with lenvatinib were enrolled. We assessed prognosis, various clinical data, body composition parameters obtained by bioelectrical impedance analysis (BIA), and handgrip strength.

**Results:**

Multivariate analysis showed that an extracellular water to total body water ratio (ECW/TBW) ≤ 0.400 at treatment initiation was associated with longer overall survival (OS), progression-free survival (PFS), and post-progression survival (PPS) (OS: hazard ratio [H0R], 4.72; 95% CI, 12.03–11.00; *P <* 0.001; PFS: HR, 2.66; 95% CI, 1.33–5.34; *P* = 0.0057; PPS: HR, 3.08; 95% CI, 1.32–7.18; *P* = 0.0093). Multivariate analysis also showed that the skeletal muscle mass index (SMI) of the arm at treatment initiation was associated with a longer PFS (HR, 2.12; 95% CI, 1.23–3.64; *P =* 0.0069). In the group with an ECW/TBW ≤ 0.400 before lenvatinib treatment, univariate analysis showed that the rate of change in only the arm SMI was associated with a longer OS and PFS.

**Conclusion:**

Body composition assessment by BIA before and after lenvatinib treatment is useful in predicting prognosis in lenvatinib-treated patients with HCC.

## Introduction

Hepatocellular carcinoma (HCC) is one of the leading causes of cancer-related death worldwide [1–3]. HCC commonly occurs in patients with chronic hepatitis or liver cirrhosis due to hepatitis B virus, hepatitis C virus, alcohol use, nonalcoholic steatohepatitis, or diabetes [4]. In recent years, the prognosis of HCC has improved due to progress in imaging technology and therapeutic strategies. However, advanced HCC still has a poor prognosis.

Lenvatinib was approved for insurance coverage in Japan in March 2018 for advanced HCC as a first-line therapy. The REFLECT study demonstrated noninferiority of lenvatinib compared with sorafenib [5]. In that study, lenvatinib had a significantly better response as assessed by the modified Response Evaluation Criteria in Solid Tumors and a longer progression-free survival (PFS) than sorafenib, although overall survival (OS) was similar between the two drugs [6].

A diagnosis of sarcopenia requires both a decrease in handgrip strength and skeletal muscle mass loss according to the diagnostic criteria of the Japan Society of Hepatology [7]. The prognosis of cirrhotic patients with sarcopenia is generally poor [8]. Previous studies have reported that in patients with HCC, sarcopenia is associated with a worse prognosis and an increased rate of cancer recurrence [9, 10]. In addition to the collective measure of sarcopenia, skeletal muscle mass loss and a decrease in handgrip strength are also independently associated with a poor prognosis [11–16]. However, the studies that led to these conclusions used single-slice computed tomography (CT), not bioelectrical impedance analysis (BIA), to measure skeletal muscle mass. In this study, we investigated the relationship between body composition before lenvatinib treatment and prognosis in patients with HCC, as well as the relationship between the rate of change in body composition during lenvatinib treatment and prognosis by measuring skeletal muscle mass with BIA.

## Materials and methods

### Patients treated with lenvatinib

The study was conducted in accordance with the Declaration of Helsinki, and the study protocol was approved by the Hiroshima University Hospital Institutional Ethics Committee (E-882). Written informed consent was obtained from each participating patient. This retrospective cohort study included 81 patients with advanced HCC who had been treated with lenvatinib at our hospital between April 2018 and April 2021. Of the 81 patients, 26 were included in our previous study [17]. These patients were considered unfit for surgery, liver transplantation, repeat locoregional therapy, repeat transcatheter arterial chemoembolization, or repeat hepatic arterial infusion chemotherapy. The inclusion criteria for treatment with lenvatinib were an Eastern Cooperative Oncology Group performance status score of ≤ 1 and Child-Pugh class A. In this study, 61 patients received lenvatinib as their first-line therapy and 20 patients had been previously treated with other molecularly targeted therapies or immune checkpoint inhibitors. The exclusion criteria for this study were (1) Child-Pugh class B or ascites, (2) a short observation period (< 1 month), (3) absence of proper image analysis, and (4) absence of body composition measurements by BIA before and after the start of lenvatinib treatment.

Tumour staging was based on the Tumour-Node-Metastasis staging system of the Liver Cancer Study Group of Japan [18]. Tumours in stage I patients had three features: (1) they were solitary, (2) they measured < 2 cm in diameter, and (3) they had no vessel invasion (n = 0). Those in stage II fulfilled two of the above three features (n = 13, 16%); those in stage III fulfilled one of the above three features (n = 27, 33%); those in stage IVA fulfilled none of the above three features, but had lymph node metastases and no distant or intrahepatic metastases (n = 15, 19%); and those in stage IVB fulfilled none of the above features, but had distant metastases (n = 26, 32%).

### Body composition analysis of patients receiving lenvatinib

Body composition was measured by BIA using Inbody 720^®^ (BioSpace Co. Ltd., Seoul, Korea). The BIA used in this study was a 4-pole, 8-point direct segment multi-frequency BIA that can evaluate not only total muscle mass and fat mass, but also muscle mass and fat mass specifically of the right arm, left arm, trunk, right leg, and left leg. The first body composition measurement was taken within 1 month of starting lenvatinib treatment, and another measurement was taken around 1 month after the end of lenvatinib treatment. We had initially planned to take body composition assessments 1 month after the start of lenvatinib treatment and every 1 to 3 months thereafter, but some patients did not cooperate, and thus, we could not be performed this analysis as planned. The skeletal muscle mass index (SMI) (kg/m^2^) was calculated by dividing the limb skeletal muscle mass (kg) by the square of the height (m^2^). Arm SMI was calculated by dividing the arm’s skeletal muscle mass (kg) by the square of the height (m^2^). Leg SMI was calculated by dividing the leg’s skeletal muscle mass (kg) by the square of the height (m^2^). According to the Japan Society of Hepatology criteria,^7^ patients with SMI values < 7.0 kg/m^2^ for males and < 5.7 kg/m^2^ for females were defined as having skeletal muscle mass loss. The BIA also measured the total fat mass and the extracellular water to total body water ratio (ECW/TBW), an oedema index. Because excessive ECW results in an oedematous state, ECW/TBW is an index that reflects the degree of oedema in a person. An ECW/TBW > 0.400 was defined as an overhydrated state [17, 19]. The rate of change in body composition was calculated by subtracting the data before (or within the first month of) lenvatinib treatment from the data after lenvatinib treatment and dividing by the data before lenvatinib treatment.

### Statistical analysis

In this study, we set the cutoff values for SMI and handgrip strength according to the sarcopenia diagnostic criteria of the Japanese Society of Hepatology, and those for arm SMI and leg SMI at the median value. Continuous variables were expressed as median (range), while categorical variables were expressed as absolute and relative frequencies. The Mann-Whitney *U* test was used to compare continuous data. Either Pearson’s chi-square test or Fisher’s exact test was used to compare significant differences in the distribution of categorical variables. The percent change in body composition was correlated using the nonparametric Spearman’s rank correlation coefficient. OS, PFS, and post-progression survival (PPS) were evaluated using Kaplan–Meier survival curves and a log-rank test. Logistic regression analysis and Cox regression analysis were carried out for multivariate analysis. Only those factors with a *P* < 0.05 in the univariate analysis were subsequently assessed in the multivariate analysis. A *P* value < 0.05 denoted a statistically significant difference. All statistical analyses were carried out with the Predictive Analytics Software R version 3.5.2.

## Results

### Patient characteristics

Patient characteristics are summarized in Table 1. The median age was 72 years. Median levels of total bilirubin, albumin (ALB), and prothrombin activity were 0.7 mg/dL, 3.6 g/dL, and 88%, respectively. Median tumour marker levels were 35.5 ng/mL for alpha fetoprotein (AFP) and 528 mAU/mL for des-gamma-carboxy prothrombin. The median handgrip strength was 31.0 kg (range: 15.3–45.8 kg) for males and 17.6 kg (range: 8.7–22.9 kg) for females. The median SMI by BIA was 7.15 kg/m^2^ (range: 5.33–9.54 kg/m^2^) for males and 5.65 kg/m^2^ (range: 4.72–6.86 kg/m^2^) for females. The median arm SMI and leg SMI by BIA, respectively, were 1.91 kg/m^2^ (range: 1.27–2.90 kg/m^2^) and 5.31 kg/m^2^ (range: 3.92–7.16 kg/m^2^) for males and 1.31 kg/m^2^ (range: 0.85–2.03 kg/m^2^) and 4.34 kg/m^2^ (range: 3.51–5.84 kg/m^2^) for females. The median ECW/TBW was 0.394 (range: 0.373–0.424). Based on the results of handgrip strength and SMI, we diagnosed 14 cases with sarcopenia according to the criteria of the Japan Society of Hepatology, and 16 cases had ECW/TBW > 0.400, indicating they were in an overhydrated state.

**Table 1:** Clinical background of patients treated with lenvatinib.

	Total	ECW/TBW ≤ 0.400	ECW/TBW > 0.400	P value
	n = 81	n = 65	n = 16	
Age, years (range)	72 (46–88)	71 (46–85)	80 (67–88)	< 0.001
Gender, Females / Males, n	17 / 64	11 / 54	6 / 10	0.090
BMI, kg/m^2^ (range)	22.8 (16.1–38.4)	22.9 (16.1–38.4)	22.6 (17.1–28.9)	0.731
Etiology, HBV / HCV / NBNC, n	16 / 25 / 40	13 / 22 / 30	3 / 3 / 10	0.503
Treatment, primary / secondary, n	61 / 20	54 / 11	7 / 9	0.003
Prothrombin activity, % (range)	88 (61–131)	88 (61–131)	89 (72–110)	0.859
Total bilirubin, mg/dL (range)	0.7 (0.3–2.1)	0.7 (0.3–1.7)	0.7 (0.3–2.1)	0.441
Albumin, g/dL (range)	3.6 (2.8–4.9)	3.7 (2.8–4.9)	3.3 (2.9–4.2)	0.024
NH_3_, μmol/L (range)	29 (10–123)	29 (10–123)	27 (10–75)	0.701
Child-Pugh score, 5 / 6, n	44 / 37	37 / 28	7 / 9	0.407
mALBI grade, 1 / 2a / 2b, n	24 / 22 / 35	22 / 17 / 26	2 / 5 / 9	0.226
SMI, females, kg/m^2^ (range)	5.65 (4.72–6.86)	5.79 (4.73–6.86)	5.30 (4.72–6.69)	0.216
SMI, males, kg/m^2^ (range)	7.15 (5.33–9.54)	7.15 (5.33–9.54)	6.97 (5.45–9.39)	0.760
Arm SMI, females, kg/m^2^, (range)	1.31 (0.85–2.03)	1.35 (1.07–2.03)	1.23 (0.85–1.32)	0.020
Arm SMI, males, kg/m^2^, (range)	1.90 (1.27–2.90)	1.90 (1.27–2.90)	1.90 (1.28–2.64)	0.846
Leg SMI, females, kg/m^2^, (range)	4.34 (3.51–5.84)	4.38 (3.66–4.93)	4.01 (3.51–5.84)	0.404
Leg SMI, males, kg/m^2^, (range)	5.31 (3.92–7.16)	5.31 (3.92–7.16)	5.36 (4.10–6.75)	0.978
Body fat percentage, females, %, (range)	31.3 (12.8–44.2)	31.3 (14.3–44.2)	32.1 (12.8–43.8)	0.660
Body fat percentage, males, %, (range)	26.5 (10.7–48.0)	25.6 (10.7–48.0)	29.0 (17.9–38.5)	0.114
Handgrip strength, females, kg (range)	17.6 (8.7–22.9)	19.8 (14.7–22.9)	14.5 (8.7–18.2)	0.023
Handgrip strength, males, kg (range)	31.0 (15.3–45.8)	32.0 (15.3–45.8)	24.5 (18.8–30.4)	0.001
Sarcopenia, + /—/ undecidable*, n	14 / 63 / 4	8 / 54 / 3	6 / 9 / 1	0.024
Main tumor size, mm (range)	33 (0–135)	24 (0–120)	41 (15–41)	0.097
Tumor occupancy, ≥ 50% / < 50, n	7 / 74	6 / 59	1 / 15	1
MVI, + / -, n	15 / 66	12 / 53	3 / 13	1
TNM staging, II / III / IVA / IVB, n	13 / 27 / 15 / 26	10 / 22 / 14 / 19	3 / 5 / 1 / 7	0.477
BCLC staging, B / C	38 / 43	30 / 35	8 / 8	0.788
AFP, ng/mL (range)	35.5 (0.5–142400)	18.8 (0.5–142400)	657.0 (1.7–39820)	0.007
DCP, mAU/mL (range)	528 (13–1083990)	201 (13–1083990)	2396 (80–287990)	0.010
EHM, + / -, n	24 / 57	17 / 48	7 / 9	0.222
LN metastasis, + / -, n	17 / 64	13 / 54	4 / 12	0.734
ECW/TBW (range)	0.394 (0.373–0.424)	0.391 (0.373–0.400)	0.403 (0.401–0.424)	< 0.001

Categorical data are represented as numbers of patients, and continuous data are represented as median and range.

*Handgrip strength cannot be measured.

AFP, alpha-fetoprotein; BCLC, Barcelona clinic liver cancer; BMI, body mass index; DCP, des-gamma-carboxy prothrombin; ECW/TBW, extracellular water/total body water; EHM, extrahepatic metastasis; HBV, hepatitis B viral infection; HCV, hepatitis C viral infection; LN, lymph node; mALBI, modified albumin-bilirubin; MVI, macroscopic vascular invasion; NBNC, non-B-non-C viral hepatitis; SMI, skeletal muscle mass index; TMN, tumor-node-metastasis classification.

### Overall survival, progression-free survival, and post-progression survival following lenvatinib treatment

**Fig 1:**
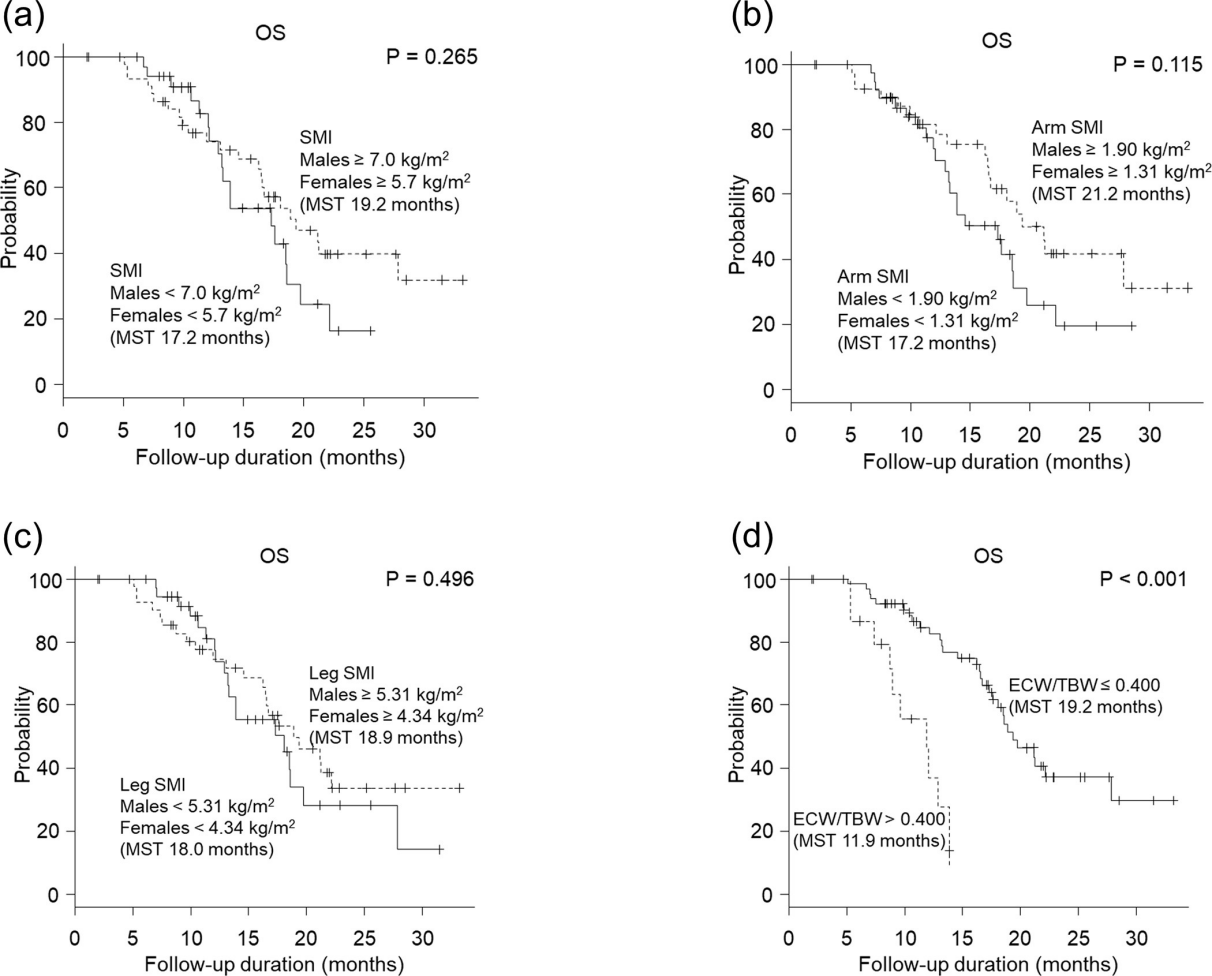
Association of each body composition measure with overall survival (OS). (a) Total skeletal muscle mass index (SMI), (b) arm SMI, (c) leg SMI, and (d) extracellular water to total body water ratio (ECW/TBW). The cutoff for SMI was 7.0 kg/m^2^ for males and 5.7 kg/m^2^ for females, which aligns with the diagnostic criteria of the Japanese Society of Hepatology. The cutoff for arm SMI and leg SMI was the median value. The cutoff for ECW/TBW was 0.400, as previously reported. (d) For the ECW/TBW, patients with an ECW/TBW of ≤ 0.400 experienced significantly longer OS than those with an ECW/TBW of > 0.400 (19.2 months vs 11.9 months, *P* < 0.001). (a—c) Total SMI, arm SMI, and leg SMI were not associated with OS (total SMI, 17.2 months vs 19.3 months, *P* = 0.265; arm SMI, 17.2 months vs 21.2 months, *P* = 0.115; leg SMI, 18.0 months vs 18.9 months, *P* = 0.496).

**Fig 2:**
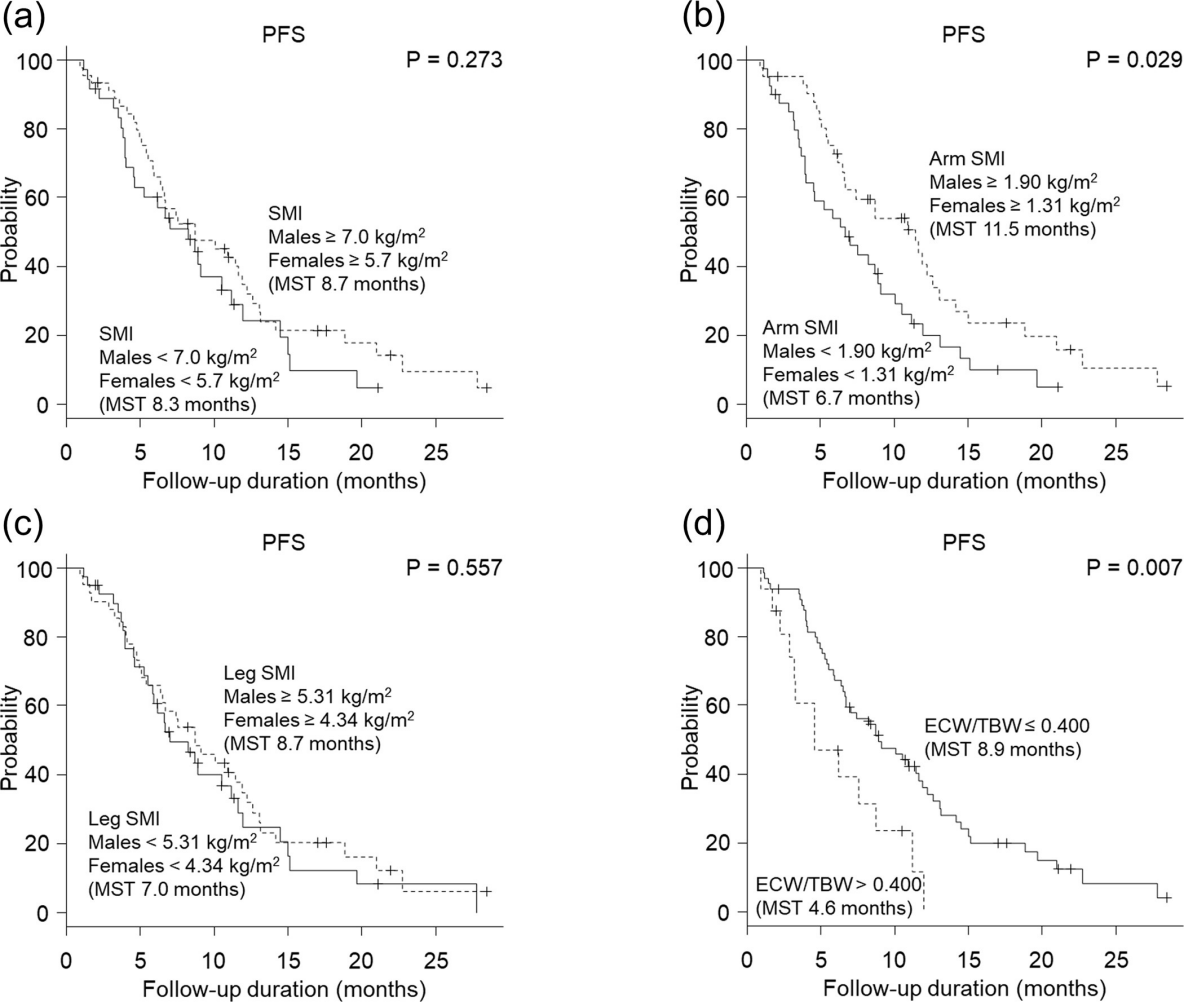
Association of each body composition measure with progression-free survival (PFS). (a) Total skeletal muscle mass index (SMI), (b) arm SMI, (c) leg SMI, and (d) extracellular water to total body water ratio (ECW/TBW). The cutoff for SMI was 7.0 kg/m^2^ for males and 5.7 kg/m^2^ for females, which aligns with the diagnostic criteria of the Japanese Society of Hepatology. The cutoff for arm SMI and leg SMI was the median value. (d) For ECW/TBW, patients with an ECW/TBW of ≤ 0.400 experienced a significantly longer PFS than those with an ECW/TBW of > 0.400 (8.9 months vs 4.6 months, *P* = 0.007). (b) For arm SMI, patients with a value above the median arm SMI experienced a significantly longer PFS than those with a value under the median arm SMI (11.9 months vs 6.7 months, *P* = 0.029). (a, c) Total SMI and leg SMI were not associated with PFS (SMI, 8.3 months vs 8.7 months, *P* = 0.273; leg SMI, 7.0 months vs 8.3 months, *P* = 0.557).

**Fig 3:**
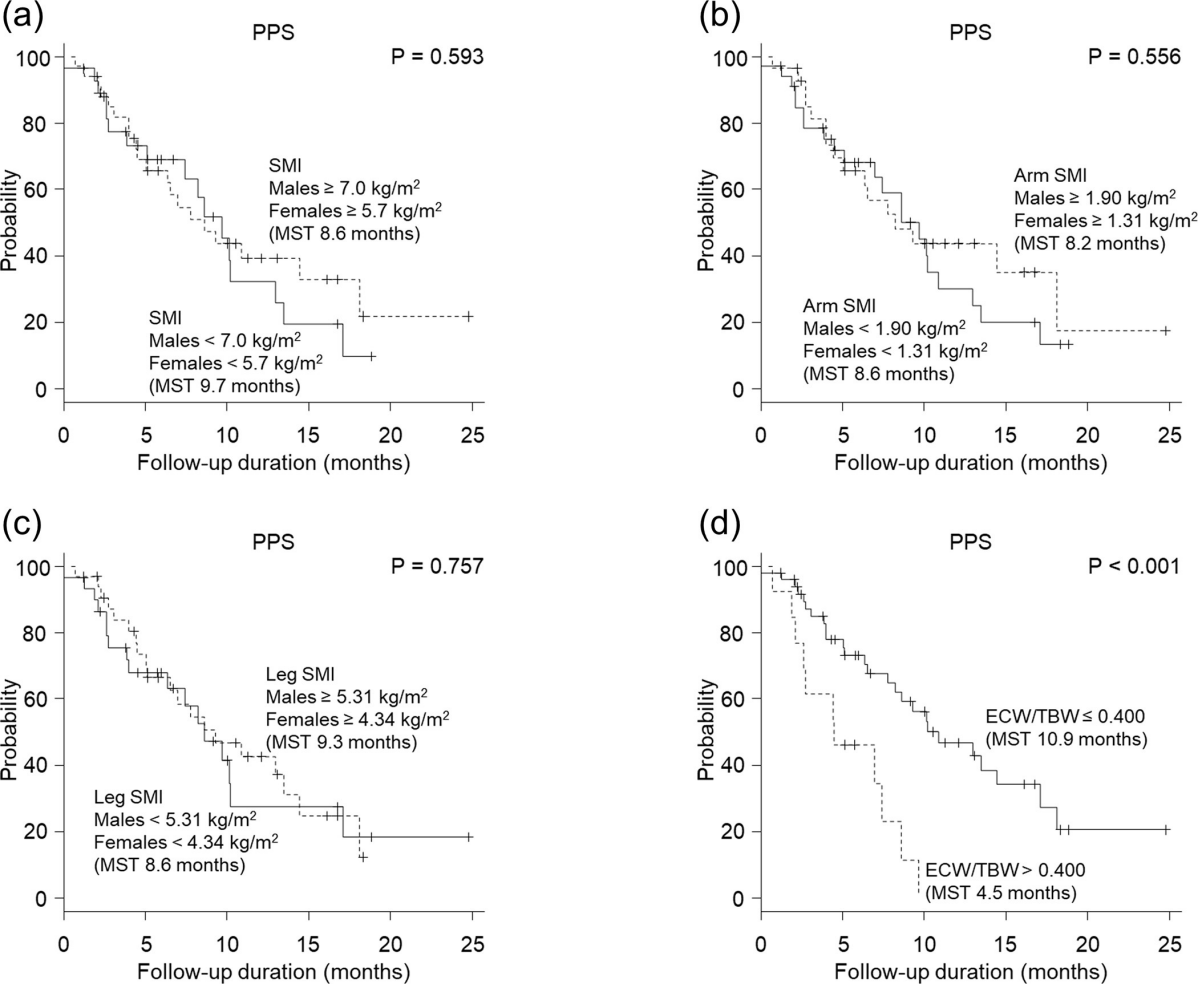
Association of each body composition measure with post-progression survival (PPS). (a) Total skeletal muscle mass index (SMI), (b) arm SMI, (c) leg SMI, and (d) extracellular water to total body water ratio (ECW/TBW). The cutoff for SMI was 7.0 kg/m^2^ for males and 5.7 kg/m^2^ for females, which aligns with the diagnostic criteria of the Japanese Society of Hepatology. The cutoff for arm SMI and leg SMI was the median value. (d) For ECW/TBW, patients with an ECW/TBW of ≤ 0.400 experienced a significantly longer PPS than those with an ECW/TBW of > 0.400 (10.9 months vs 4.5 months, *P* < 0.001). (a—c) Total SMI, arm SMI, and leg SMI were not associated with PPS (total SMI, 8.6 months vs 9.7 months, *P* = 0.593; arm SMI, 8.2 months vs 8.6 months, *P* = 0.556; leg SMI, 9.3 months vs 8.6 months, *P* = 0.757).

Figs 1 to 3 show comparisons, based on body composition, for OS, PFS, and PPS in HCC patients. Patients with an ECW/TBW ≤ 0.400 experienced a significantly longer OS than those with an ECW/TBW > 0.400 (*P* < 0.001; Fig 1). Univariate analysis showed that alanine aminotransferase (ALT) ≤ 30 IU/L, ALB > 3.5 g/dL, macroscopic vascular invasion (MVI), AFP ≤ 400 ng/mL, and an ECW/TBW ≤ 0.400 were significant factors contributing to an extension of OS. Multivariate analysis showed that MVI (hazard ratio [HR], 2.53; 95% CI, 1.19–5.40; *P =* 0.016), AFP < 400 ng/mL (HR, 2.03; 95% CI, 1.06–3.89; *P =* 0.033), and ECW/TBW ≤ 0.400 (HR, 4.72; 95% CI, 2.03–11.00; *P <* 0.001) at initiation of treatment were significant and independent determinants for an extended OS (Table 2). SMI and sarcopenia were not associated with OS.

**Table 2:** Factors associated with overall survival treated with lenvatinib.

			Univariate analysis			Multivariate analysis		
		n	Median (months)	95% CI	P Value	HR	95% CI	P Value
Age	< 70 years	25	21.2	13.0—NA				
	≥ 70 years	56	17.5	13.9–19.7	0.182			
Gender	Females	17	17.5	12.0—NA				
	Males	64	18.6	14.5–21.2	0.686			
Treatment	1st line	61	18.9	16.6–22.1				
	2nd line	20	13.5	9.7–21.2	0.111			
BMI	< 22 kg/m^2^	33	17.2	13.2—NA				
	≥ 22 kg/m^2^	48	18.6	16.2–21.2	0.805			
Platelet	< 100,000 /μL	19	17.5	12.0–21.2				
	≥ 100,000 /μL	62	18.5	16.2–27.8	0.358			
ALT	≤ 30 IU/L	47	19.7	16.5—NA		Reference		
	> 30 IU/L	34	16.5	11.3–19.3	0.020	1.60	0.82–3.11	0.165
ALB	≤ 3.5 g/dL	33	17.2	12.9–18.9		Reference		
	> 3.5 g/dL	48	19.7	16.2—NA	0.047	0.74	0.39–1.41	0.363
NH_3_	≤ 32 μmol/L	48	17.5	13.8–27.8				
	> 32 μmol/L	31	18.5	13.0–22.1	0.702			
Tumor occupancy	< 50%	74	18.5	16.2–21.2				
	≥ 50%	7	9.9	5.4—NA	0.254			
MVI	-	66	19.3	16.6–27.8		Reference		
	+	15	12	7.0–18.9	0.002	2.53	1.19–5.40	0.016
AFP	< 400 ng/mL	53	19.3	17.5—NA		Reference		
	≥ 400 ng/mL	28	13	9.9–16.5	0.003	2.03	1.06–3.89	0.033
DCP	< 1000 mAU/mL	48	18.9	16.5–22.1				
	≥ 1000 mAU/mL	33	12.9	9.9—NA	0,231			
EHM	-	57	18.6	16.5–27.8				
	+	24	13.0	9.0–21.2	0.064			
LN metastasis	-	64	18.9	16.5–22.1				
	+	17	14.5	10.4–27.8	0.133			
SMI	Females, < 5.7 kg/m^2^, Males, < 7.0 kg/m^2^	36	17.2	12.9–19.7				
	Females, ≥ 5.7 kg/m^2^, Males, ≥ 7.0 kg/m^2^	45	19.3	16.2—NA	0.265			
Arm SMI	Females, < 1.31 kg/m^2^, Males, < 1.90 kg/m^2^	40	17.2	12.9–18.6				
	Females, ≥ 1.31 kg/m^2^, Males, ≥ 1.90 kg/m^2^	41	21.2	16.5—NA	0.115			
Leg SMI	Females, < 4.34 kg/m^2^, Males, < 5.31 kg/m^2^	40	18.0	12.9–19.7				
	Females, ≥ 4.34 kg/m^2^, Males, ≥ 5.31 kg/m^2^	41	18.9	16.2—NA	0.496			
Handgrip strength	Females, < 18 kg, Males, < 26 kg	23	12.9	9.7–19.7				
	Females, ≥ 18 kg, Males, ≥ 26 kg	54	18.9	16.5–27.8	0.052			
Sarcopenia	-	63	18.6	16.5–22.1				
	+	14	12.9	7.0—NA	0.298			
ECW/TBW	< 0.400	65	19.2	17.2–27.8		Reference		
	≥ 0.400	16	11.9	7.4–13.8	< 0.001	4.72	2.03–11.00	< 0.001

AFP, alpha-fetoprotein; ALB, albumin; ALT, alanine aminotransferase; BMI, body mass index; CI, confidence interval; DCP, des-gamma-carboxy prothrombin; ECW/TBW, extracellular water/total body water; EHM, extrahepatic metastasis; HR, hazard ratio; LN, lymph node; mALBI, modified albumin-bilirubin; MVI, macroscopic vascular invasion; NA, not applicable; SMI, skeletal muscle mass index.

Patients with an ECW/TBW ≤ 0.400 and arm SMI ≥ 1.31 kg/m^2^ for females and ≥ 1.90 kg/m^2^ for males experienced a significantly longer PFS than those with an ECW/TBW > 0.400 and arm SMI < 1.31 kg/m^2^ for females and < 1.90 kg/m^2^ for males (*P* = 0.00671 and 0.029; Fig 2). Univariate analysis showed that ALB > 3.5 g/dL, NH_3_ ≤ 32 μmol/L, MVI, arm SMI ≥ 1.31 kg/m^2^ for females and ≥ 1.90 kg/m^2^ for males, and an ECW/TBW ≤ 0.400 were significant factors contributing to an extension of PFS. Multivariate analysis showed that NH_3_ ≤ 32 μmol/L (HR, 2.36; 95% CI, 1.28–4.34; *P =* 0.0060), MVI (HR, 2.13; 95% CI, 1.11–4.09; *P =* 0.023), arm SMI ≥ median value (HR, 2.12; 95% CI, 1.23–3.64; *P =* 0.0069), and ECW/TBW ≤ 0.400 (HR, 2.66; 95% CI, 1.33–5.34; *P* = 0.0057) at initiation of treatment were significant and independent determinants for extension of PFS (Table 3). Sarcopenia was not associated with PFS.

**Table 3:** Factors associated with progression free survival treated with lenvatinib.

			Univariate analysis			Multivariate analysis		
		n	Median (months)	95% CI	P Value	HR	95% CI	P Value
Age	< 70 years	25	7.5	5.4–11.9				
	≥ 70 years	56	8.7	5.6–11.6	0.750			
Gender	Females	17	7.6	4.0–11.6				
	Males	64	8.7	6.4–11.5	0.508			
Treatment	1st line	61	8.9	6.4–12.3				
	2nd line	20	8.7	4.6–10.5	0.116			
BMI	< 22 kg/m^2^	33	7.6	4.6–10.5				
	≥ 22 kg/m^2^	48	8.7	6.4–11.9	0.515			
Platelet	< 100,000 /μL	19	6.7	4.1–10.9				
	≥ 100,000 /μL	62	8.7	6.2–12.0	0.088			
ALT	≤ 30 IU/L	47	8.7	5.6–10.9				
	> 30 IU/L	34	7.6	5.9–12.0	0.915			
ALB	≤ 3.5 g/dL	33	6.5	4.8–9.1		Reference		
	> 3.5 g/dL	48	10.1	6.6–12.6	0.027	0.99	0.55–1.76	0.968
NH_3_	≤ 32 μmol/L	48	10.1	6.7–12.0		Reference		
	> 32 μmol/L	31	6.5	4.6–9.1	0.017	2.36	1.28–4.34	0.006
Tumor occupancy	< 50%	74	8.2	6.4–11.2				
	≥ 50%	7	10.9	0.9—NA	0.821			
MVI	-	66	8.9	6.7–11.9		Reference		
	+	15	5.1	3.9–6.2	0.024	2.13	1.11–4.09	0.023
AFP	< 400 ng/mL	53	10.1	6.7–12.0				
	≥ 400 ng/mL	28	6.2	4.6–9.1	0.231			
DCP	< 1000 mAU/mL	48	8.9	6.5–12.3				
	≥ 1000 mAU/mL	33	6.6	4.8–11.5	0.637			
EHM	-	57	8.9	6.4–11.6				
	+	24	6.6	5.0–9.1	0.245			
LN metastasis	-	64	8.7	6.2–11.5				
	+	17	6.7	4.6–12.6	0.955			
SMI	Females, < 5.7 kg/m^2^, Males, < 7.0 kg/m^2^	36	8.2	4.6–10.5				
	Females, ≥ 5.7 kg/m^2^, Males, ≥ 7.0 kg/m^2^	45	8.7	6.4–11.9	0.273			
Arm SMI	Females, < 1.31 kg/m^2^, Males, < 1.90 kg/m^2^	40	6.7	4.0–9.1		Reference		
	Females, ≥ 1.31 kg/m^2^, Males, ≥ 1.90 kg/m^2^	41	11.5	6.6–12.6	0.029	0.47	0.27–0.81	0.007
Leg SMI	Females, < 4.34 kg/m^2^, Males, < 5.31 kg/m^2^	40	7.0	5.6–11.2				
	Females, ≥ 4.34 kg/m^2^, Males, ≥ 5.31 kg/m^2^	41	8.7	5.3–11.9	0.557			
Handgrip strength	Females, < 18 kg, Males, < 26 kg	23	6.7	3.2–12.0				
	Females, ≥ 18 kg, Males, ≥ 26 kg	54	8.7	6.2–11.6	0.419			
Sarcopenia	-	63	8.7	6.2–10.9				
	+	14	6.7	3.2–19.7	0.980			
ECW/TBW	< 0.400	65	8.9	6.6–11.9		Reference		
	≥ 0.400	16	4.6	2.9–8.7	0.007	2.66	1.33–5.34	0.006

AFP, alpha-fetoprotein; ALB, albumin; ALT, alanine aminotransferase; BMI, body mass index; CI, confidence interval; DCP, des-gamma-carboxy prothrombin; ECW/TBW, extracellular water/total body water; EHM, extrahepatic metastasis; HR, hazard ratio; LN, lymph node; mALBI, modified albumin-bilirubin; MVI, macroscopic vascular invasion; NA, not applicable; SMI, skeletal muscle mass index.

Patients with an ECW/TBW ≤ 0.400 experienced a significantly longer PPS than those with an ECW/TBW > 0.400 (*P* < 0.001; Fig 3). Univariate analysis showed that ALT ≤ 30 IU/L, AFP ≤ 400 ng/mL, extrahepatic metastasis, lymph node metastasis, and ECW/TBW ≤ 0.400 were significant factors contributing to extension of PPS. Multivariate analysis showed that ALT ≤ 30 IU/L (HR, 2.10; 95% CI, 1.07–4.13; *P =* 0.031) and ECW/TBW ≤ 0.400 (HR, 3.08; 95% CI, 1.32–7.18; *P* = 0.0093) at initiation of treatment were significant and independent determinants for extension of PPS (Table 4). SMI and sarcopenia were not associated with PPS.

**Table 4:** Factors associated with post progression survival treated with lenvatinib.

			Univariate analysis			Multivariate analysis		
		n	Median (months)	95%CI	P Value	HR	95% CI	P Value
Age	< 70 years	22	9.2	4.0—NA				
	≥ 70 years	40	8.6	6.4–10.9	0.381			
Gender	Females	15	7.8	2.8–13.5				
	Males	47	9.7	5.1–14.5	0.574			
Treatment	1st line	44	10.2	6.5–14.5				
	2nd line	18	7.0	2.6–9.3	0.159			
BMI	< 22 kg/m^2^	26	9.7	7.5–13.0				
	≥ 22 kg/m^2^	36	7.8	4.5–14.5	0.915			
Platelet	< 100,000 /μL	18	9.3	2.6–14.5				
	≥ 100,000 /μL	44	8.6	6.4–13.0	0.728			
ALT	≤ 30 IU/L	38	10.2	7.0—NA		Reference		
	> 30 IU/L	24	6.4	2.8–10.1	0.015	2.10	1.07–4.13	0.031
ALB	≤ 3.5 g/dL	28	10.1	2.8–13.5				
	> 3.5 g/dL	34	8.6	6.4—NA	0.298			
NH_3_	≤ 32 μmol/L	33	7.8	4.5–10.1				
	> 32 μmol/L	27	10.9	4.4–17.1	0.444			
Tumor occupancy	< 50%	57	9.2	7.0–13.0				
	≥ 50%	5	4.4	2.8—NA	0.461			
MVI	-	51	9.7	6.3–13.5				
	+	11	7.5	2.8–8.6	0.324			
AFP	< 400 ng/mL	40	10.9	7.0–17.1		Reference		
	≥ 400 ng/mL	22	7.5	3.9–8.6	0.035	1.49	0.72–3.08	0.281
DCP	< 1000 mAU/mL	38	10.2	7.8–14.5				
	≥ 1000 mAU/mL	24	7.2	4.0–13.5	0.543			
EHM	-	41	10.2	8.2–17.1		Reference		
	+	21	5.0	2.1–7.0	0.016	1.99	0.99–4.01	0.054
LN metastasis	-	47	10.1	8.2–14.5		Reference		
	+	15	5.0	2.6–7.5	0.025	1.42	0.62–3.28	0.410
SMI	Females, < 5.7 kg/m^2^, Males, < 7.0 kg/m^2^	28	9.6	5.1–13.0				
	Females, ≥ 5.7 kg/m^2^, Males, ≥ 7.0 kg/m^2^	34	8.6	4.5–18.1	0.593			
Arm SMI	Females, < 1.31 kg/m^2^, Males, < 1.90 kg/m^2^	34	8.6	5.1–10.9				
	Females, ≥ 1.31 kg/m^2^, Males, ≥ 1.90 kg/m^2^	28	8.2	4.4–18.1	0.556			
Leg SMI	Females, < 4.34 kg/m^2^, Males, < 5.31 kg/m^2^	30	8.6	4.0–10.2				
	Females, ≥ 4.34 kg/m^2^, Males, ≥ 5.31 kg/m^2^	32	9.3	5.1–14.5	0.757			
Handgrip strength	Females, < 18 kg, Males, < 26 kg	16	7.5	3.9–13.0				
	Females, ≥ 18 kg, Males, ≥ 26 kg	43	9.3	6.4–17.1	0.218			
Sarcopenia	-	50	8.6	6.5–13.5				
	+	9	7.5	0—NA	0.492			
ECW/TBW	< 0.400	49	10.9	7.6–17.1		Reference		
	≥ 0.400	13	4.5	2.1–8.6	< 0.001	3.08	1.32–7.18	0.009

AFP, alpha-fetoprotein; ALB, albumin; ALT, alanine aminotransferase; BMI, body mass index; CI, confidence interval; DCP, des-gamma-carboxy prothrombin; ECW/TBW, extracellular water / total body water; EHM, extrahepatic metastasis; HR, hazard ratio; LN, lymph node; mALBI, modified albumin-bilirubin; MVI, macroscopic vascular invasion; NA, not applicable; SMI, skeletal muscle mass index.

### Amount of change in body composition during lenvatinib treatment

**Fig 4:**
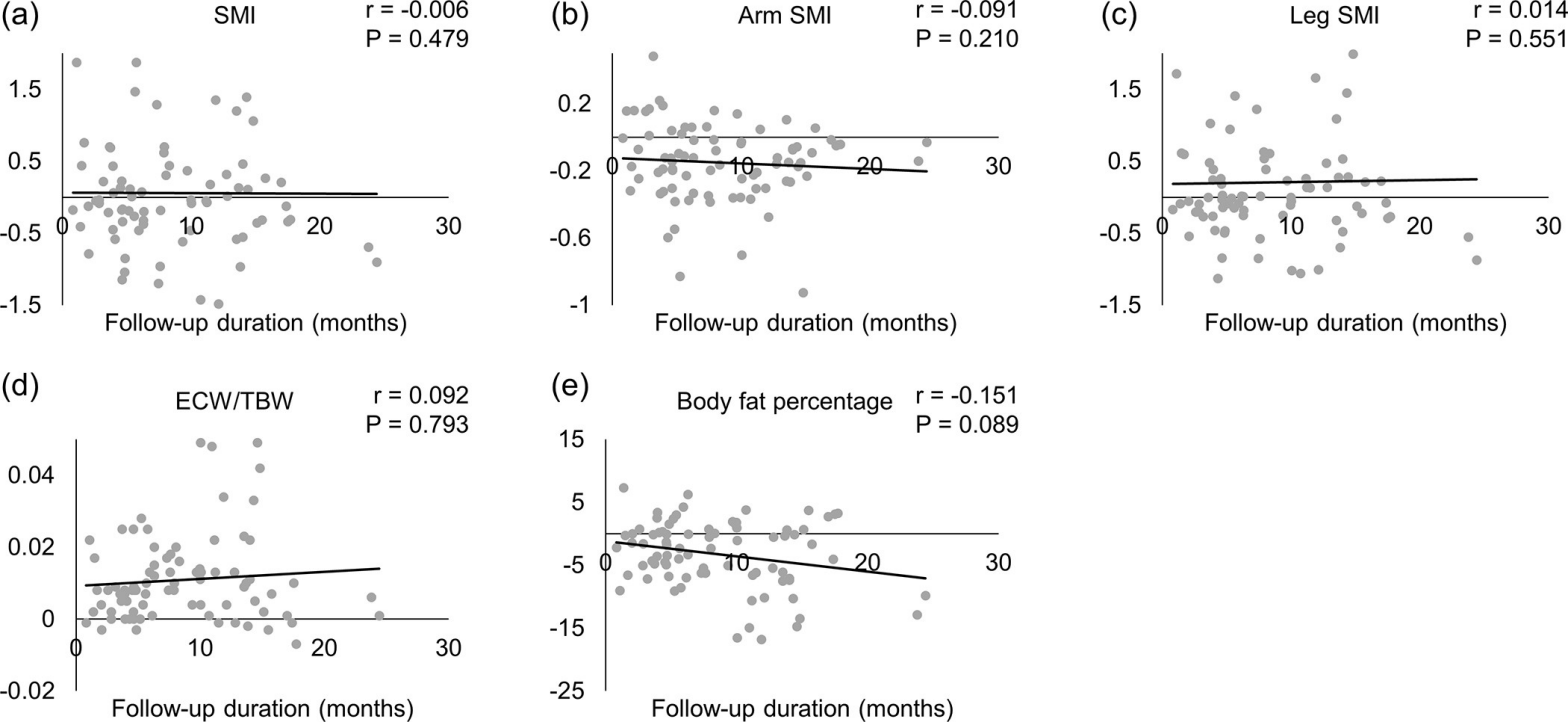
Scatter plots and approximate lines of changes in body composition and duration. (a) Total skeletal muscle mass index (SMI), (b) arm SMI, (c) leg SMI, (d) extracellular water to total body water ratio (ECW/TBW), and (e) body fat percentage. There was no correlation between the follow-up duration and the amount of change in body composition. The horizontal axis shows the time from the start of lenvatinib treatment to the day of the second body composition measurement, and the dots show the percentage change in body composition. (b) The arm SMI decreased in many cases and (d) the ECW/TBW increased in many cases.

**Fig 5:**
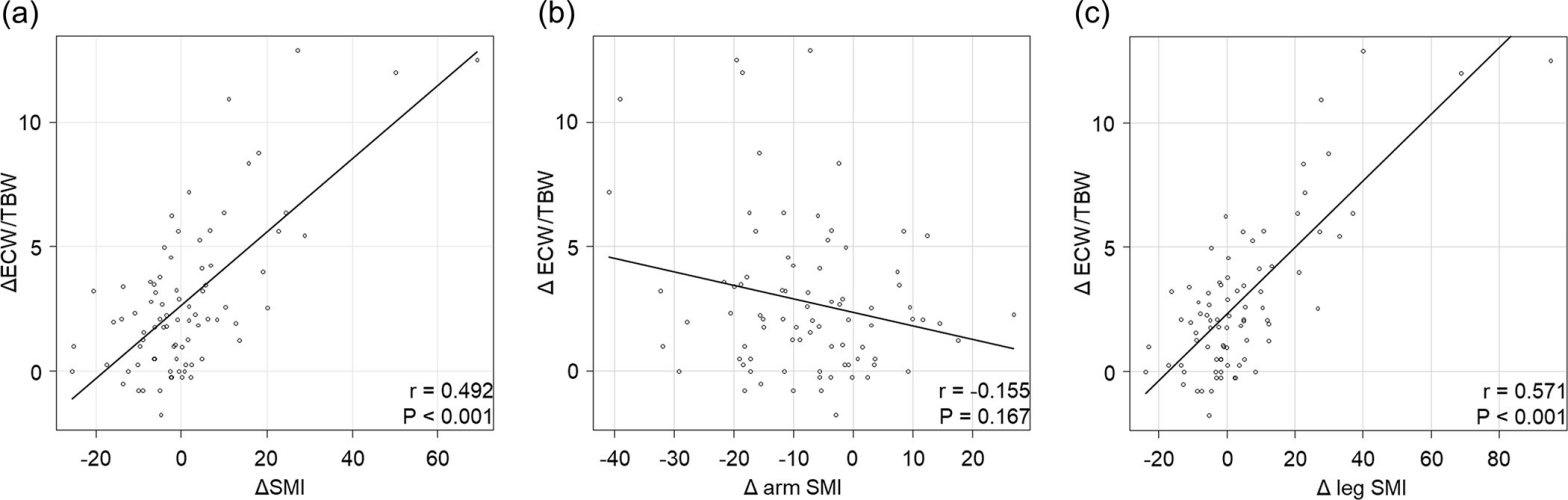
Correlation between the rate of change in ECW/TBW and that for each SMI measure. (a) Total SMI, (b) arm SMI, and (c) leg SMI. (b) The ECW/TBW and arm SMI were not correlated with the rate of change (r = −0.155, *P* = 0.167). (a) The rate of change of the ECW/TBW and total SMI and (c) that of ECW/TBW and leg SMI were positively correlated (r = 0.492, *P* < 0.001 and r = 0.571, *P* < 0.001).

The median duration from the start of lenvatinib treatment to the 2nd body composition assessment after starting lenvatinib treatment was 5.9 months (range: 0.8–24.4 months). The correlation between the amount of change in body composition and the duration of lenvatinib treatment is shown in Fig 4. Total SMI and leg SMI measured by BIA showed an increase after treatment in skeletal muscle mass in 36 and 40 cases, respectively. Therefore, we examined the correlation between the rate of change in ECW/TBW and the rate of change in SMI and leg SMI and found that it was significantly correlated (r = 0.492, *P* < 0.001 and r = 0.586, *P* < 0.001; Fig 5).

### Association with rate of change in body composition and prognosis

**Fig 6:**
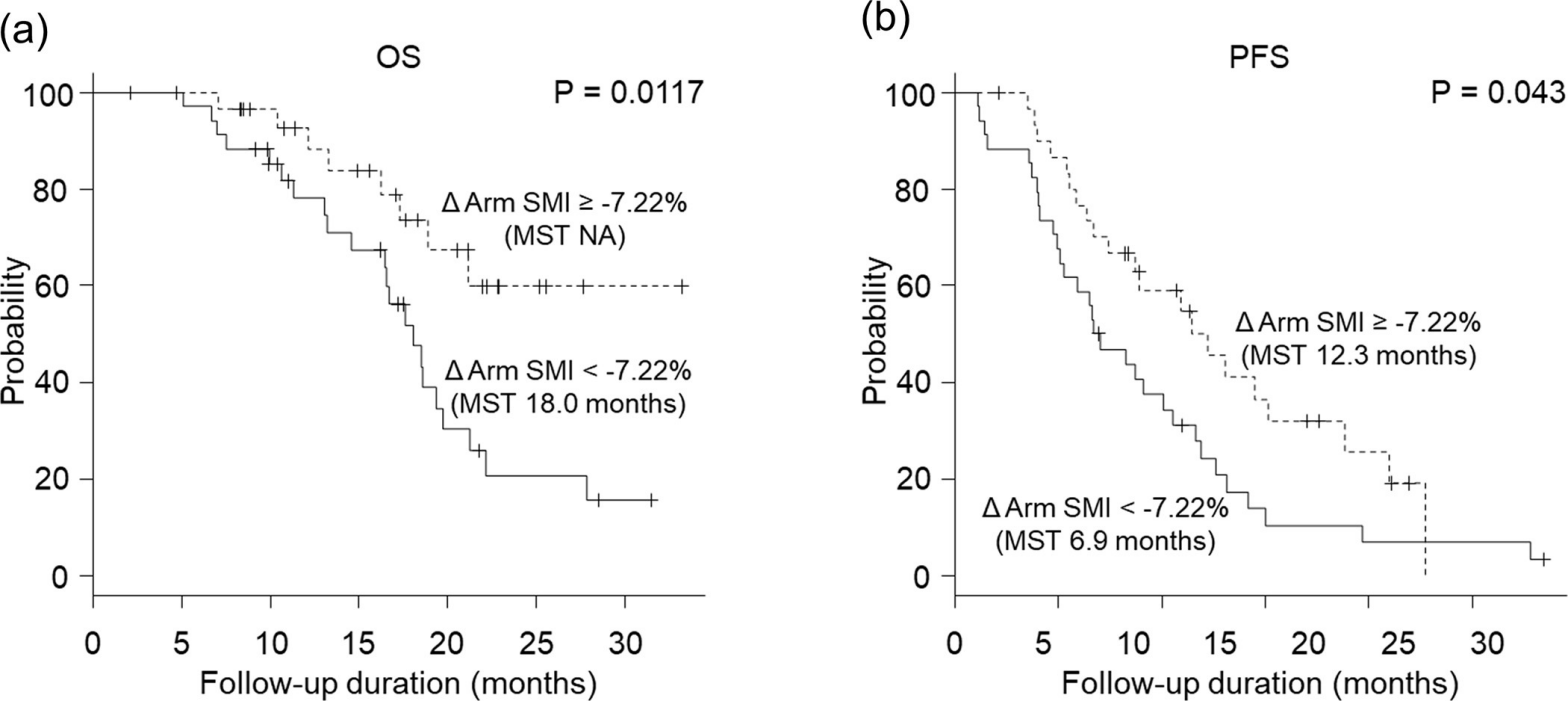
Association between the rate of change in body composition and prognosis. The group with ECW/TBW ≤ 0.400 before lenvatinib treatment with a rate of change in the arm skeletal muscle mass index (SMI) above the median had a significantly longer (a) overall survival (OS) (data not available vs 18.0 months, *P* = 0.0117) and (b) progression-free survival (PFS) (12.3 months vs 6.9 months, *P* = 0.043) than the group with a rate of change in the arm SMI below the median.

The median rates of change in arm SMI, leg SMI, ECW/TBW, and body fat percentage were −7.22%, −0.12%, −2.09%, and −9.57%, respectively. We set the median as the cutoff value and examined the relationship between the rate of change in body composition and prognosis, but none of the results were significantly different. Next, we examined the association between the rate of change in body composition and prognosis in a subgroup of patients with an ECW/TBW < 0.400 before lenvatinib treatment, as this group had a better prognosis. The arm SMI change > −7.22% group had a longer OS and PFS compared to the change < −7.22% group (Fig 6). There were no significant differences between prognosis and the rates of change in leg SMI, ECW/TBW, and body fat percentage.

## Discussion

**Fig 7:**
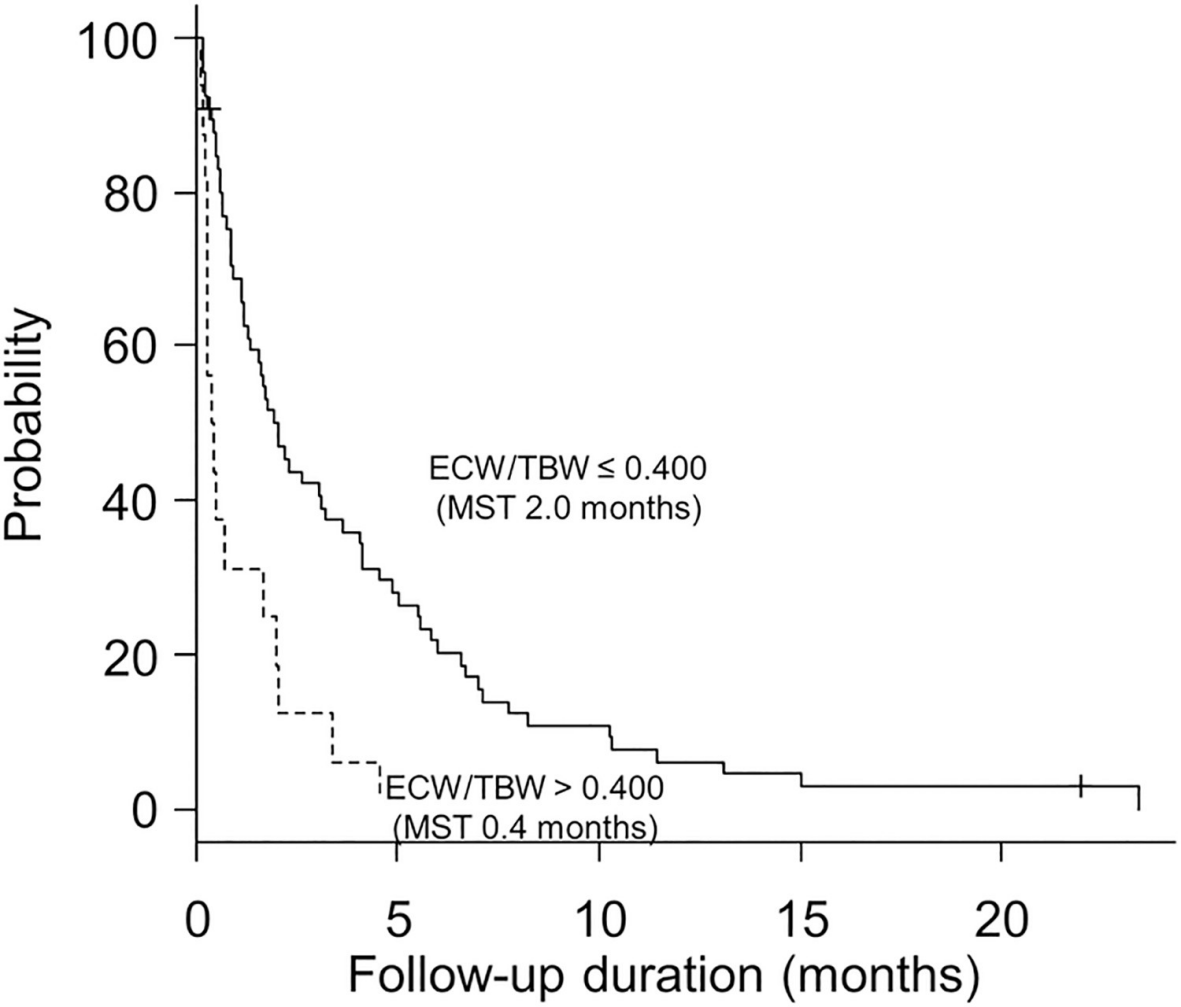
Association of extracellular water to total body water ratio with the duration of first dose reduction or drug withdrawal. The cutoff for extracellular water to total body water ratio (ECW/TBW) was 0.400, as previously reported. Patients with an ECW/TBW of > 0.400 experienced significantly shorter duration of first dose reduction or drug withdrawal than those with an ECW/TBW of ≤ 0.400 (0.4 months vs 2.0 months, P < 0.001).

In HCC treatment, only our previous report has examined body composition measurement in BIA [17], and there are no reports on body composition changes in BIA. ECW/TBW is an index that reflects oedema, and a high ECW/TBW indicates increased fluid retention [19, 20]. Previously, we reported that patients with an ECW/TBW > 0.400 have a shorter duration until reduction or withdrawal of lenvatinib and have difficulty maintaining the relative dose intensity, though PFS did not differ based on ECW/TBW [17]. In this study, the number of cases included was higher than in the previous study, offering a greater statistical power. In addition, OS and PPS were also evaluated in addition to PFS due to the longer follow-up duration. In this study, we found that an ECW/TBW > 0.400 before lenvatinib treatment was associated with shorter OS, PFS, and PPS. Notably, ECW/TBW increases as cirrhosis progresses, with the median value of ECW/TBW being 0.393 in Child–Pugh class A patients, 0.402 in Child–Pugh class B patients, and 0.405 in Child–Pugh class C patients [21]. Based on this, patients with an ECW/TBW > 0.400 were speculated to have a poor liver functional reserve, elevated blood levels of lenvatinib, and earlier onset of adverse events. In this study, the median duration to first dose reduction or drug withdrawal was 1.6 months. The median duration to first dose reduction or drug withdrawal in the group with ECW/TBW > 0.400 was 0.6 months, which was significantly shorter than that in the group with ECW/TBW < 0.400, which was 2.0 months. The short duration of the first dose reduction or drug withdrawal may suggest a decrease in relative dose intensity (Fig 7). Therefore, an ECW/TBW > 0.400 may have contributed to the shortened OS and PFS because these patients were less likely to continue lenvatinib treatment. With this result, we reconfirmed that ECW/TBW is useful in screening Child–Pugh class A patients for tolerability of lenvatinib treatment.

Previous reports have shown that loss of skeletal muscle mass is associated with a worse prognosis in HCC [11–16]. Other reports indicate that skeletal muscle mass loss is a prognostic factor in lenvatinib treatment [15, 16]. There are some methods to measure SMI: single-slice CT, BIA, and dual energy X-ray absorptiometry (DXA). Previous studies have used single-slice CT to measure skeletal muscle mass [11–16]. Single-slice CT is a common test, but is difficult to repeat due to radiation exposure. DXA also involves radiation exposure, but the low dose allows for repeat examinations and regional body composition assessment. BIA is simple, non-invasive and repeatable as DXA. In this study, we used BIA to measure body composition because BIA has the greatest advantage over single-slice CT or DXA in that there is no radiation exposure no matter how many times it is measured. When we measured SMI using BIA, however, we found that SMI was not associated with prognosis in this study, as previous reports using CT have found. This may be due to some limitations of BIA. Namely, the chemical composition of fat-free masses (i.e., water, proteins, etc.) have considerable inter- and intraindividual variability because of changes in fat-free mass that occur during disease states [22]. In addition, one report showed a decrease in skeletal muscle mass measured by BIA after haemodialysis [23]. Therefore, SMI may be overestimated due to oedema when it is measured by BIA. In fact, in our study, there was a positive correlation between the rate of change in ECW/TBW and the rate of change in SMI after lenvatinib treatment, and thus, SMI before treatment may have been affected by the oedema rate. Moreover, another study reported that the arm SMI value was better associated with prognosis compared to the leg SMI value in patients with cirrhosis [24]. When SMI was examined separately for the upper and lower limbs in this study, the group with a low SMI of the arm had a shorter PFS than the group with a high SMI of the arm. These data suggest that SMI as measured by BIA may be higher than it really is when there is fluid overload, with leg SMI being more susceptible to oedema. In this study, we examined the correlation between the rate of change of ECW/TBW for arm SMI and leg SMI. ECW/TBW showed a positive correlation with the leg SMI, but not with the arm SMI. Therefore, to accurately assess SMI during lenvatinib treatment in HCC patients, it should be assessed with arm SMI.

We previously reported that tyrosine kinase inhibitor treatment for HCC caused skeletal muscle loss [25]. In this study, we found that the rate of change in arm SMI had an impact on prognosis in the group with an ECW/TBW < 0.400. Several factors are associated with skeletal muscle mass loss. A previous study showed that reasons for skeletal muscle depletion in HCC include decreased physical activity and poor nutrition due to disease progression and the adverse effects of treatment, as well as increased expression of inflammatory cytokines in patients with cancer [9]. Furthermore, sarcopenia is associated with alterations in the phosphoinositide PI3k/Akt/mTOR signalling pathway [26–29], which is associated with muscle protein synthesis. Both sorafenib and lenvatinib, used for HCC treatment, inhibit VEGFR-mediated signalling and carnitine transporters, which may suppress signalling in the downstream PI3k/Akt/mTOR pathway, leading to subsequent skeletal muscle mass loss [30]. Skeletal muscle mass loss caused by molecularly targeted drugs is associated with poor prognosis in non–small cell lung cancer [31, 32], but this has not been reported in HCC until now. This is the first report that skeletal muscle mass loss during lenvatinib treatment is associated with a worse prognosis in HCC. When checking the rate of change of skeletal muscle mass in BIA, it is important to check it using the rate of change of arm SMI.

This study has several limitations, such as the small sample size, retrospective design, and short-term nature. Also, the timing for the second assessment of body composition varied between patients. In future studies, we expect the number of cases available for study to increase, the follow-up duration to be lengthened, and the timing of measuring body composition after lenvatinib treatment to be matched.

In conclusion, body composition measurement using BIA is simple, non-invasive, repeatable, and does not involve radiation exposure. Additionally, BIA can measure not only skeletal muscle mass, but also body fat percentage and ECW/TBW. Since arm SMI is associated with PFS, and ECW/TBW is associated with OS, PFS, and PPS, assessing body composition with BIA before lenvatinib treatment is extremely useful as a prognostic factor. We also found that a great decrease in arm SMI was associated with prognosis in patients with an ECW/TBW ≤ 0.400 before treatment. Our data indicate that it is important to assess body composition before as well as during lenvatinib treatment.
